# Criação de Modelos Embriológicos Cardíacos para Impressão 3D para Ensino de Anatomia e Embriologia

**DOI:** 10.36660/abc.20220632

**Published:** 2023-03-24

**Authors:** Davi Shunji Yahiro, Juliana Cadilho da Silva Abrantes, D’Angelo Carlo Magliano, Claudio Tinoco Mesquita

**Affiliations:** 1 Universidade Federal Fluminense Niterói RJ Brasil Universidade Federal Fluminense (UFF), Niterói, RJ – Brasil; 2 Hospital Universitário Antônio Pedro EBSERH UFF Niterói RJ Brasil Health, Science & Education Lab - Hospital Universitário Antônio Pedro - EBSERH – UFF, Niterói, RJ – Brasil; 3 Programa de Pós Graduação em Ciências Cardiovasculares Universidade Federal Fluminense Niterói RJ Brasil Programa de Pós Graduação em Ciências Cardiovasculares da Universidade Federal Fluminense (UFF), Niterói, RJ – Brasil; 4 Núcleo de Pesquisa em Morfologia e Metabolismo Niterói RJ Brasil Núcleo de Pesquisa em Morfologia e Metabolismo, Niterói, RJ – Brasil

**Keywords:** Medicina/embriologia, Coração/anatomia, Imagem tridimensional/tendências

## Introdução

Os estudantes de medicina têm uma grande dificuldade em visualizar as estruturas embriológicas e entender o desenvolvimento morfológico. Estudos relatam que os alunos comumente consideram a Embriologia como uma disciplina difícil e não se sentem confiantes com o conhecimento obtido.^1 , 2^ O aprendizado de embriologia tradicional envolve a leitura de livros e interpretação de imagens planas, o que dificulta a percepção espacial e o entendimento do processo de formação embriológica.

A tecnologia de modelagem tridimensional (3D) comumente oferece serviços para a engenharia, arquitetura, desenvolvimento de jogos e filmes.^3^ O desenvolvimento de modelos 3D consiste em criar e ligar vértices para formar malhas poligonais.^3^ A criação destas malhas permite a visualização em perspectiva, além de poder ser colorida, texturizada e animada pelo designer, e, posteriormente, poder ser impressas. A criação de modelos 3D pode ser muito vantajosa na Embriologia, uma vez que há estudos que mostram a tecnologia 3D como uma ferramenta auxiliadora para o ensino de anatomia e para o planejamento de cirurgias complexas.^4 - 7^

Levando em consideração a complexidade do desenvolvimento do coração e a dificuldade apresentada pela maioria dos estudantes no aprendizado da embriologia cardíaca, este trabalho relata o desenvolvimento de modelos 3D para facilitar o aprendizado médico, buscando demonstrar o looping cardíaco e a septação atrial e ventricular, pontos críticos do desenvolvimento do coração.

## Métodos

Trata-se de um estudo descritivo e observacional. Relatamos os resultados da criação dos modelos 3D para o ensino da embriologia cardíaca, considerando a evidência presente na literatura sobre os benefícios do uso da tecnologia 3D na compreensão da embriologia cardíaca.

O trabalho iniciou com uma revisão da literatura sobre a embriologia cardíaca para a criação dos modelos com imagens de livros textos da área, apostilas de ensino médico e artigos científicos. Por meio do Blender ®, software de modelagem 3D de código aberto, foram criadas malhas, seguindo as referências obtidas, reproduzindo modelos embriológicos cardíacos. Esses modelos foram criados, texturizados e animados num computador do tipo PC com placa gráfica de desempenho normal.

Posteriormente, dos 15 modelos criados, nove foram impressos através da impressora AnyCubic Kobra, com filamento PLA 1.75 mm na cor branca. A duração da impressão para cada modelo foi de aproximadamente 2,5 horas.

Os arquivos estão disponíveis para download gratuito no endereço eletrônico: https://github.com/daviyahiro/cardiac-embryological-models.

## Resultados

Foram criados 15 modelos os quais mostram: junção dos tubos cardíacos, looping cardíaco, formação dos coxins endocárdicos, septação atrial, forame primário, forame oval e septação ventricular.

Além disso, com esses modelos, foi possível criar duas animações que demonstram o passo a passo do looping cardíaco e a septação atrial, semelhantemente às imagens de materiais didáticos, porém com profundidade. As animações, salvadas em .mp4, podem ser encontradas no mesmo endereço eletrônico dos modelos. Posteriormente, os modelos foram impressos para melhorar a experiência de ensino, permitindo a interação concreta do objeto.

**Figura 1:**
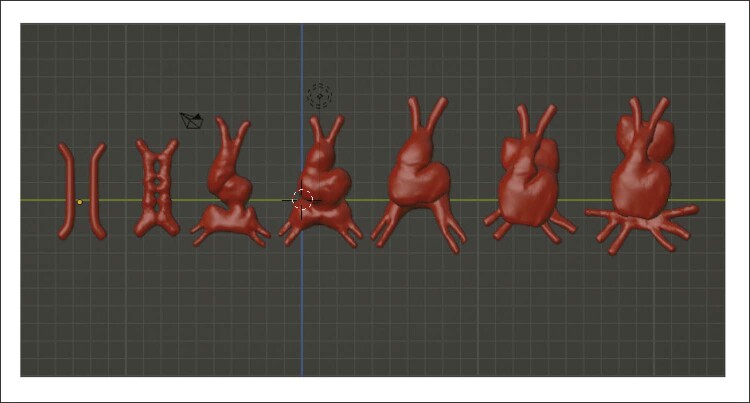
– Etapas do dobramento cardíaco.

Na Figura 1 , é possível ver a dobra do coração, desde a junção dos tubos, formação da curvatura em C e finalmente dobra, que também podem ser manipulados para alterar a perspectiva em um software adequado.

**Figura 2:**
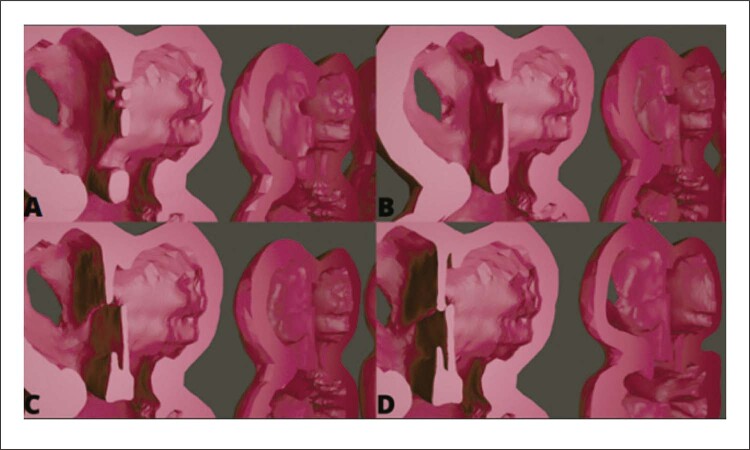
– Etapas da septação atrial.

A Figura 2 apresenta a formação do septo atrial seguindo as suas devidas etapas, mostrando o septo primário, septo secundário, forame primário, forame secundário e forame oval. Apesar das imagens apresentar o mesmo ângulo, elas podem ser movidas de acordo com a necessidade do usuário.

**Figura 3:**
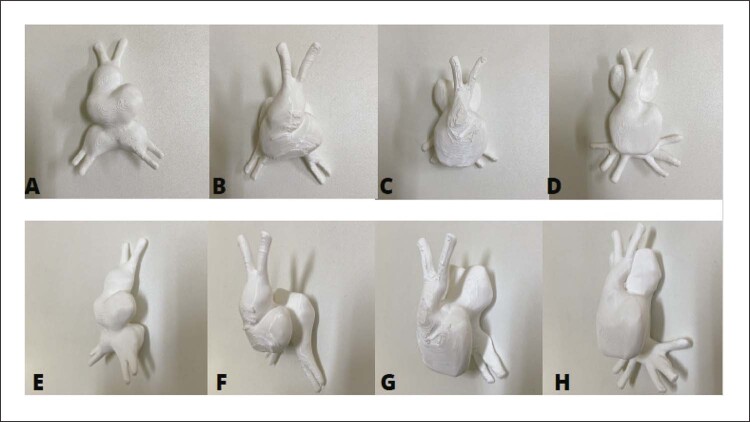
– Modelos impressos do dobramento.

Nas Figuras 3 e 4 , estão os nove modelos, em estágios diferentes, impressos para a utilização no ensino.

**Figura 4:**
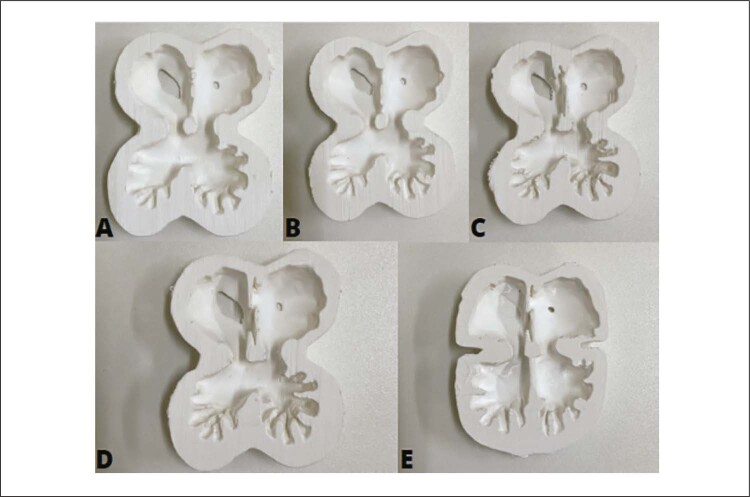
– Modelos da septação atrial.

## Discussão

O correto entendimento do desenvolvimento cardíaco é passo fundamental para identificação e manejo das diversas malformações congênitas do sistema circulatório.^8^

Os modelos 3D mostram uma perspectiva e profundidade que não é possível em livros didáticos ou imagens. Esses modelos são de fácil acesso para visualização, uma vez que os arquivos são salvos em formato STL e podem ser manipulados por um celular, em aplicativos gratuitos como o ViewSTL®, ou um computador em sites online, possibilitando até mesmo importação para a tecnologia de Realidade Virtual, transformando-os em uma experiência mais rica.

Além disso, os modelos impressos possuem baixo custo, já que se utilizam filamentos de plástico PLA ou ABS para impressão. Dessa forma, é possível ver com detalhes auxiliando no ensino de graduação ou comunicação com os pacientes e familiares sobre as malformações cardíacas.

Há relatos na literatura de melhora no ensino através de modelos feitos de biscuit ou massa de modelar,^9^ porém as impressões 3D podem ser reproduzidas em maior quantidade e com menos desgaste. Além disso, as impressões oferecem uma possível solução para a dificuldade em obter peças anatômicas,^10^ que restringe algumas instituições de ensino.

Os modelos também são úteis na criação de animações e vídeos em que mostram a formação do septo atrial em uma visão de perspectiva, dando melhor entendimento da sequência do desenvolvimento embrionário. Estudos mostram que o uso de materiais visuais complementa o ensino e auxiliam no engajamento do aluno na disciplina de Embriologia,^1 , 2^ especialmente no âmbito da embriologia cardíaca.^11 , 12^

## Conclusões

Os modelos 3D apresentam vantagens quanto a sua reprodutibilidade e possibilidade de disponibilidade online para uso em diversas instituições. Esta técnica é muito versátil com a possibilidade de utilização em animações e criação de vídeos que auxiliem no aprendizado. A criação de modelos embriológicos de outras estruturas embriológicas ou até mesmo de modelos de doenças congênitas pode contribuir ainda mais na educação médica. Espera-se que os modelos 3D criados possibilitem a melhora na educação da embriologia cardíaca através da experiência visual e tátil que elas permitem.
