# Supplementary material for: Criação de Modelos Embriológicos Cardíacos para Impressão 3D para Ensino de Anatomia e Embriologia
Source: Arq Bras Cardiol. 2023 Mar 24;120(4):e20220632. [Article in Portuguese] doi: 10.36660/abc.20220632 (PMC10124573; doi:10.36660/abc.20220632)
Supplement: Translation

# Translation: Creation of Cardiac Embryological Models for 3D Printing to Teach Anatomy and Embryology

**Authors:** Davi Shunji Yahiro, Juliana Cadilho da Silva Abrantes, D’Angelo Carlo Magliano, Claudio Tinoco Mesquita

**Affiliations:** 1 Brazil Universidade Federal Fluminense (UFF), Niterói, RJ – Brazil; 2 Brazil Health, Science & Education Lab - Hospital Universitário Antônio Pedro - EBSERH – UFF, Niterói, RJ – Brazil; 3 Brazil Programa de Pós Graduação em Ciências Cardiovasculares da Universidade Federal Fluminense (UFF), Niterói, RJ – Brazil; 4 Brazil Núcleo de Pesquisa em Morfologia e Metabolismo, Niterói, RJ – Brazil

**Keywords:** Medicine/embriology, Heart/anatomy, Imaging, Three-Dimensional/trends

## Introduction

Medical students often struggle to visualize embryonic structures and understand morphological development. Studies report that students commonly consider Embryology to be a difficult subject and do not feel confident in the knowledge they have obtained.^1 , 2^Traditional embryology learning involves reading books and interpreting flat images, interfering with spatial perception and understanding of embryonic development.^2^

Three-dimensional (3D) modeling technology commonly provides engineering, architecture, gaming, and film animation services.^3^The development of 3D models consists of creating voxels and connecting vertices to form polygonal meshes.^3^These meshes allow for perspective visualization and the designer’s ability to color, texture, and animate them, and they can be printed. The creation of 3D models can be very advantageous in Embryology, as studies show 3D technology as an aiding tool for teaching anatomy and planning complex surgeries.^4 - 7^

Considering the complexity of heart development and the difficulty presented by most students in learning cardiac embryology, this paper reports the development of 3D models to facilitate medical learning, aiming to demonstrate cardiac looping and atrial and ventricular septation, important points of heart development.

## Methods

This is a descriptive and observational study. We report the results of creating 3D models for teaching cardiac embryology, considering the evidence present in the literature on the benefits of using 3D technology in understanding cardiac embryology.

The work began with a literature review on cardiac embryology for reference of the models based on images from textbooks, medical teaching materials, and scientific articles. Using Blender ®, an open-source 3D modeling software, the meshes were created, following the references obtained, reproducing cardiac embryological models. These models were created, textured, and animated on a PC with a normal performance graphics card.

Subsequently, after the creation of 15 models created, nine of them were printed using the AnyCubic Kobra printer, with 1.75 mm PLA filament in white. Each model took approximately 2.5 hours to print.

Files can be found to download and visualization at: https://github.com/daviyahiro/cardiac-embryological-models.

## Results

Fifteen models were created to demonstrate: cardiac tubes fusion, cardiac looping, formation of endocardial cushions, atrial septation, *foramen primum, foramen ovale,* and ventricular septation.

In addition, with these models, it was possible to create two animations demonstrating the step-by-step cardiac looping and atrial septation, like the images of didactic materials, but with depth. The animations were saved in .mp4 and can be found at the same electronic address as the models. Subsequently, the models were printed to improve the teaching experience, allowing for concrete interaction with the object.

In figure 1 , it is possible to observe the looping of the heart from the junction of the tubes, the formation of the C-shaped bend, and finally, looping, which can also be manipulated to change the perspective in software that manipulates .stl files.

**Figure 1:** – Steps of cardiac looping.

Figure 2 shows the atrial septum formation following its proper stages, showing the *septum primum, septum secundum, foramen primum, foramen secundum,* and *foramen ovale.* Although the images present the same angle, they can be moved according to the user.

**Figure 2:** – Stages of atrial septation.

Figures 3 and 4 show the nine printed models, in different stages, printed for demonstration in the classroom and manipulation for the students.

**Figure 3:** – Printed models of cardiac looping.

**Figure 4:** – Printed models of atrial septation.

## Discussion

The correct understanding of cardiac development is a fundamental step for identifying and managing various congenital malformations of the circulatory system.^8^

3D models provide a perspective and depth impossible in textbooks or images. These models are easy to access for interaction since the files are saved in STL format and can be manipulated by a cell phone, in free applications such as ViewSTL®, or a computer in online sites, even allowing importation to the Virtual Reality technology, creating a richer experience.

In addition, the printed models have low cost since 3D printing requires plastic filaments such as PLA or ABS. Thus, it is possible to see it in detail, helping in teaching or even in patient communication and family members about cardiac malformations.

There are reports in the literature of improved teaching through models made of biscuits or modeling clay,^9^however, 3D prints can be reproduced in greater quantity and with high reproducibility. In addition, prints offer a possible solution to the difficulty in obtaining anatomical specimens,^10^which restricts some educational institutions.

The models are also useful in creating animations and videos that show the atrial septum formation from a perspective view, providing a better understanding of the sequence of embryonic development. Studies show that using visual materials complements teaching and aids in students’ engagement in the discipline of Embryology,^1 , 2^especially in the context of cardiac embryology.^11 , 12^

## Conclusions

3D models offer advantages in reproducibility and possible online availability for use in several institutions. This technique is very versatile because it allows animations and video creation to assist in learning and training. Creating embryological models of other embryonic structures or congenital diseases may contribute even more to medical education. It is expected that the created 3D models can improve the education of cardiac embryology through the visual and tactile experience they allow.
